# Quality of life in coeliac disease: qualitative interviews to develop candidate items for the Coeliac Disease Assessment Questionnaire

**DOI:** 10.2147/PROM.S149238

**Published:** 2018-07-04

**Authors:** Helen Crocker, Crispin Jenkinson, Michele Peters

**Affiliations:** Health Services Research Unit, Nuffield Department of Population Health, University of Oxford, Oxford, UK, michele.peters@dph.ox.ac.uk

**Keywords:** coeliac disease, quality of life, patient-reported outcome measure, item development, qualitative, content validity, conceptual framework

## Abstract

**Background:**

Coeliac-specific measures have been criticized for not complying with current guidance on the development of patient-reported outcome measures (PROMs). The aim of this study was to develop a measure to assess health-related quality of life in adults with coeliac disease (CD), in accordance with current guidance for PROM development.

**Methods:**

In-depth qualitative interviews were conducted with adults with CD. A thematic analysis was undertaken to develop a coding framework. All interviews were analyzed according to this framework. Interviewing continued until data saturation was achieved. Candidate items were developed on the basis of the interview findings.

**Results:**

The analysis revealed 6 themes: 1) symptoms, 2) gluten-free diet, 3) emotional health, 4) impact on activities, 5) relationships, and 6) financial issues. Data saturation was reached after 8 interviews, but a total of 23 interviews were conducted to include a wide enough range of diverse participants. From the themes, 64 candidate items (9 for symptoms, 15 for emotional health, 16 for gluten-free diet, 7 for relationships, 12 for impact on activities, and 5 for financial issues) were developed to form the first draft of the Coeliac Disease Assessment Questionnaire (CDAQ).

**Conclusion:**

The 64 items reflect all the issues of importance to people with CD. Next, these items will be pretested and refined to lead to a shorter draft version of the CDAQ before it is administered in a survey to produce a final version with subscales.

## Introduction

Coeliac disease (CD) is a chronic autoimmune condition triggered by the consumption of gluten, a protein found in wheat, barley, and rye. The only treatment currently available is a gluten-free diet, which is burdensome, restrictive, and challenging to adhere to.^1^–^3^ CD can lead to complications such as osteoporosis, autoimmune diseases, and vitamin D and iron deficiencies,^4^,^5^ and there is a small increased risk of mortality.^6^ The gluten-free diet can cause difficulties in various aspects of daily life, including traveling, shopping, and eating meals outside of the home.^7^ Furthermore, it is recognized that a diet is unlikely to ever be completely gluten free (eg, due to issues of cross-contamination), and therefore, the diet may not be able to fully control the symptoms and disease activity.^8^ Clinical trials are currently underway to test alternative treatments,^9^ but despite these potentially new treatments, the diet will remain important as it is likely that the first medication entering the market will be supplementary to, rather than a substitute for, the gluten-free diet.^10^

Interest in measuring patient-reported outcomes such as health-related quality of life (HRQoL) has increased over the last decade. Patient-reported outcome measures (PROMs) present a unique opportunity to gain insight into the patients’ views, which may not overlap with clinical outcomes or biomedical markers.^11^ PROMs have been proposed as potential endpoints for clinical trials in CD^9^ and as a method to capture the patients’ illness experience in a systematic way, which may help to direct care and improve clinical outcomes.^11^

Patient outcomes or HRQoL has predominantly been assessed using generic measures, most commonly the 36-item Short Form Health Survey (SF-36).^12^,^13^ More recently, disease-specific measures have been developed including the Coeliac Disease Questionnaire (CDQ)^14^ and the Coeliac Disease Quality of Life (CD-QoL) survey.^15^ Symptom checklists that are patient reported such as the disease-specific Celiac Symptom Index^16^ have also been developed. To be considered useful in clinical trials, PROMs should have been developed according to the guidelines of the US Food and Drugs Administration (FDA).^17^ While the FDA guidelines were developed with labeling claims in mind, they are regarded as best practice for the development of PROMs regardless of their intended use.^18^ A systematic review identified 4 candidate PROMs for use in clinical trials and concluded that none of the existing CD-specific PROMs currently meet FDA regulations.^19^

Due to the limitations of existing CD-specific measures, the overall aim of this study was to develop a new PROM, in line with best practice guidelines, for assessing outcomes in adults with CD. The intention was to develop a measure that included all aspects of CD that impact HRQoL in 1 measure as identified by people with CD. To develop a new PROM, interviews or focus groups with participants from the target population must contribute toward item generation.^17^ As well as being considered good practice, this is “essential” for establishing content validity,^20^,^21^ ie, ensuring the measure is comprehensive. This article presents the results from the qualitative interviews and the development of candidate items. The aim of the qualitative interviews was to gain in-depth understanding of the impact of CD on HRQoL from the perspective of adults with the condition. The findings from the interviews were then used to develop candidate items for a new PROM for adults with CD.

## Methods

Ethics approval was received from the University of Oxford Central University Research Ethics Committee (REC References: MSD/IDREC/C1/2012/38).

### Recruitment

Participants were recruited through Coeliac UK, a charity for people with CD, which has ~60,000 members, and also via referral (ie, snowballing) from those recruited via the charity. People with CD were eligible to participate if they were aged 18 years or older and self-reported a diagnosis of CD. Participants were recruited from 3 geographical regions in England (Yeovil, Oxford, and Birmingham). These regions were selected to maximize variability between rural and urban areas, counties, and more and less socially deprived areas. To capture a wide range of experience, a maximum variation sampling strategy was taken, with variation sought across gender, age, ethnicity, duration since diagnosis, and clinical presentation. Twenty members were invited from each region by Coeliac UK. The invitation included a cover letter from Coeliac UK, a participant information sheet, a consent form, and a prepaid return envelope. Those interested in taking part were asked to return a completed written consent form to the research team. An initial discussion with people who returned the consent form was held over the telephone. This telephone discussion aimed to gather some preliminary information about participants (such as gender, age, and time since diagnosis) to aid the recruitment of a diverse sample and to start building rapport with the participants. During the telephone call, potential participants were asked to confirm their diagnosis of CD. As a part of the interview, participants were asked to complete a short demographics form (eg, age, employment status, and ethnicity) and to confirm their diagnosis of CD and the length of time since diagnosis.

A review of the demographic and disease characteristics of respondents who had returned consent forms highlighted limited, or an absence of, representation of certain groups, ie, younger members, members diagnosed more recently, members belonging to black and minority ethnic groups, and members living in central urban locations. In an attempt to include individuals from these groups, a further 30 members were selected from Coeliac UK’s database and invited to participate.

### Interview

A semistructured interview guide (Table 1) was developed on the basis of current literature by HC and MP. HC conducted a mock interview to test the interview guide. The interview guide was modified as necessary as the interviews progressed to allow fuller exploration of emerging themes. All interviews were audio recorded.

Interviews took place at the participant’s convenience, in their home, workplace, or the University of Oxford, and were conducted by HC between June and October 2012. The majority of interviews were conducted with only the participant present, although 5 participants were interviewed with the spouse present for at least part of the interview. All participants gave written informed consent. Interviews continued until data saturation was reached, ie, the point at which no new information is obtained. The achievement of data saturation in PROM development is important to ensure that the relevant constructs are sufficiently mapped.^22^ A data saturation table was constructed, showing the emergence of themes across interviews.^22^

### Data analysis and item development

Interviews lasted between 50 minutes and 2 and a half hours and were transcribed verbatim by a professional transcriber or HC. All transcripts were checked against the original recordings by HC.

The transcripts were coded and analyzed in NVivo 9 using a thematic approach.^23^ A bottom up approach was used to ensure that the themes stemmed from the content of the interviews. An initial coding framework was developed by coding the transcripts of the first 4 interviews and evolved further as the analysis progressed, with new codes added as new themes arose and similar or overlapping codes collapsed. HC and MP each coded 2 transcripts and compared their codes to develop, and where appropriate modify, the coding framework. Once the coding framework was developed, all transcripts were systematically coded using this framework by HC. The main themes and subthemes were identified, discussed by all the authors, and contributed to the conceptual framework underpinning HRQoL in CD.

Candidate items were drafted for each of the themes emerging from the analysis. The drafting of items was an iterative process, and items were developed, amended, and deleted by consensus between all the authors until a final draft list, together with questionnaire instructions and response options, was achieved for pretesting.

## Results

### Participants

Forty-one (46% of those invited) Coeliac UK members returned a consent form. Single interviews were undertaken until data saturation was achieved. Consequently, 23 adults with CD (15 women and 8 men) were interviewed, of whom 19 adults recruited via Coeliac UK and 4 adults recruited through snowball sampling. It was expected that more women would be recruited than men, as CD is more common in women.^24^,^25^ Participants were aged between 29 and 90 years and diagnosed for between 3 months and 40 years (Table 2).

### Themes

The participants discussed about a range of HRQoL factors that were impacted by their CD. These factors revealed the following 6 themes (each with subthemes; Table 3): 1) symptoms, 2) gluten-free diet, 3) emotional health, 4) impact on activities, 5) relationships, and 6) financial issues. Data saturation is illustrated in Table 4, with the table showing both when an issue (code) was first discussed and how many participants discussed the issue. As data saturation was reached after 8 interviews, not all 41 respondents were interviewed. Twenty-three interviews were conducted, ie, 15 interviews past the point of data saturation to include a wide enough range of diverse participants (eg, to include additional younger participants) and to allow sufficient depth to be achieved.

#### Symptoms

Symptoms were experienced mainly prior to diagnosis, but some participants also explained that symptoms could occur postdiagnosis as a result of gluten consumption. Symptoms often impacted on participants’ daily activities, such as commuting and ability to focus on tasks. The main symptoms experienced were gastrointestinal, such as loose bowel movements, diarrhea, abdominal bloating and discomfort, stomach pain, stomach cramps, nausea, and vomiting. An 82-year-old man, diagnosed for 40 years, described his reaction to consuming gluten:

Just violently sick and bad diarrhoea that bad like, it comes on quick and you’re, you’re just stuck with it. [Participant 1]

Additional symptoms reported by the majority of participants included problems with energy, weight, and pain. A 70-year-old man, diagnosed for 15 years, described his tiredness:

All I was able to do was to maybe take the washing out of the washing machine and put it, hang it up or go down to the shop just down the road, get a bit of shopping then I’d come back here and I’d spend the rest of the day asleep in the chair literally, just asleep and the only things that stimulated me physically at all were sugar and alcohol. [Participant 13]

Furthermore, participants described a wide range of other symptoms or health consequences including poor concentration or “brain fog”, anemia, osteoporosis, rashes, and mouth ulcers. These additional symptoms or health consequences varied across participants, with sometimes only 1 or 2 participants describing a specific symptom.

#### Gluten-free diet

The gluten-free diet is currently the only treatment for CD, and participants elaborated on the challenges faced by the need to follow it. Participants encountered practical difficulties in relation to obtaining gluten-free food, worries about cross-contamination, and poor palatability of gluten-free substitute foods. When participants were able to purchase substitute foods, many were disappointed with their taste and texture, particularly bread, describing it as “absolutely vile” and “too crumbly to make a sandwich”.

Difficulties in obtaining gluten-free food were encountered in shops such as supermarkets and when eating away from home. Participants needed to place trust in other people to provide them with gluten-free food and when they did not feel confident that the food was gluten-free, it left them with a dilemma as to whether to eat the food or not as a 65-year-old female participant explained.

I tell you what’s awkward when people [um]; [name] brought me back a cake that […] and I just think what baking powder did she use, she’s not got the baking powder. And I suppose if I’m feeling, if I’m on a good phase I’ll think, ‘Oh I’ll risk it, it’s fine, I know what it is.’ […] So things like that, so you’re put in an awkward social situation because something, something is probably OK but may not be. I do find that slightly awkward, because I’d actually rather not have it. [Participant 15]

A key difficulty was cross-contamination, ie, the contamination of gluten-free food with gluten. This was a particular problem when eating out in restaurants and cafes but could also occur in the home. Some participants went to great lengths to prevent cross-contamination, such as using separate toasters and cooking utensils, while others were less strict with preventing cross-contamination. The precautions taken to avoid cross-contamination appeared to be related to the severity of symptoms experienced when gluten had been consumed. A 87-year-old man commented:

I shouldn’t really use the same toaster, but um, we just keep it clean […] I’m not so worried, about the um, slight contamination, which um, someone who’s got it [CD] really badly would need to. [Participant 3]

#### Emotional health

CD also impacted on emotional health, with participants describing various worries and concerns. While 1 concern was whether family members, particularly children and grandchildren, would develop the condition, the majority of the concerns related to the participant’s own health, for example, developing symptoms after consuming gluten and developing associated conditions such as osteoporosis. Other negative emotions that participants experienced were depression, exclusion from attending social activities, and isolation. Feeling isolated was described as feeling “alone”, such as “an outsider”, and “at sea” and occurred when participants took part in an activity but were somewhat removed, for example, eating a packed lunch while others purchased food from a cafe. A 59-year-old woman explained her feelings of isolation:

I think the greatest thing is that you feel like an outsider, nearly all the time when it comes to it and you dread anybody bringing up food or going out for a meal or going to somebody’s house or let’s go to a café and have, a cake or something 9 times out of 10 you can’t do that, so when a group of people say oh yea lets go out for a meal you think oh god here we go again. [Participant 8]

Participants disliked receiving attention because of their CD, with many finding this embarrassing, while also disliking having to “make a fuss” to ensure that their gluten-free diet was catered for. Participants adopted strategies to avoid drawing attention to their CD, such as accepting offers of sweets or biscuits “for later” to avoid having to explain their condition.

#### Impact on activities

The activities that were impacted by CD were wide ranging and included work, social activities, travel, and holidays. Impacts on activities due to symptoms primarily occurred prior to diagnosis and also impeded activities following diagnosis, due to gluten consumption or the need to follow a gluten-free diet.

Participants described that their ability to attend work and perform was impacted by gastrointestinal symptoms and tiredness. This could lead to arriving late for work, taking days off, and, in 2 extreme cases, resigning from their job. A 71-year-old woman describes how CD impacted her work.

To make a decision […] I think, I’d actually make quite a point of that decision because that does affect you quite profoundly and certainly workwise, you know, you shilly shally around and you don’t want to commit yourself to writing this letter or, or something because you know it’s hard to make the decision whether you’ve, whether you’ve covered all the points. [Participant 4]

Participants’ ability to travel (eg, to work), go on holiday, and participate in social activities were also impeded. Some participants described feeling excluded when socializing with colleagues, as they were unable to eat foods brought into share, or were not catered for events. A 38-year-old female participant explained how CD impacted on her ability to travel and ultimately her work:

I’d have stomach-ache, umm and lots of trips to the bathroom, I mean it got to a ridiculous stage where I couldn’t even get to work, I couldn’t make the journey from home to work without having to go to the bathroom. [Participant 20]

In an attempt to reduce the impact of CD on activities, participants developed strategies to maintain their gluten-free diet and cope with symptoms, for example, eating before attending a party, taking food on holiday, and carrying spare underwear to cope with symptoms. In some circumstances, participants chose not to attend activities, or selected alternative activities, due to difficulties explaining their dietary needs and avoiding becoming ill. A 32-year-old female commented:

I’m a bit scared about travelling, not around Europe maybe, but I was half thinking of going to Morocco next year and half of me’s afraid to go because of my stomach, just in case there’s no there’s not a lot of option and I don’t want to be diarrhoea and sick on holidays and just not feeling well. [Participant 23]

#### Relationships

Most participants found their family and friends to be supportive and understanding of their CD, which was demonstrated by, for example, their diet being accommodated during visits or choosing restaurants known to offer gluten-free options. Family and friends were sometimes able to help participants navigate their gluten-free diet, when they did not feel able to do so themselves, as explained by a recently diagnosed 29-year-old woman:

Sometimes I don’t feel like I want to, you know I don’t want to force the issue but because [husband’s name] is like my wingman so every time I feel like I can’t mention it [the GFD] he’ll say ‘you know this is just the way it has to be, and you know I’m sorry it’s a faff but, that’s just it’. [Participant 21]

However, this was not the case with all relationships, with many participants recalling at least some negative experiences, such as being called “faddy” and “too fussy”. Such comments were frustrating for participants, and a 59-year-old woman remarked:

You kind of wish that if you ate it [gluten] you’d collapse down on the floor clasping at your stomach and get whisked off to hospital because then people would see a reaction. [Participant 8]

Participants also recognized the impact of their CD on family and friends, noting that family members needed to take precautions with preparing suitable food. Several participants also discussed the general disruption to family life, resulting from issues such as the need for all family members to take precautions to avoid cross-contamination and, in a few cases, family members also followed a gluten-free diet within the home. Participants described instances where other people had assured them that the food was gluten free, but they were subsequently unwell, which suggested that the food had contained gluten. Therefore, placing trust in others to provide gluten-free food could prove challenging with 1 participant describing it as “a bit nerve-wracking because you’re just putting your health in someone else’s hands and trusting them to know” (Participant 21).

#### Financial issues

The theme of financial issues was less complex but nonetheless of great importance to participants. The main financial concern for participants was directly linked to the gluten-free diet due to the high cost of gluten-free substitute foods such as bread and pasta. This caused a great deal of frustration, with participants calling prices “extortionate”, “outrageous”, and “unfair”. Several participants felt guilty that family and friends had bought expensive gluten-free alternatives for them to eat when visiting. The majority of participants did not report any other financial issues; however, for 2 participants, the financial impact went beyond the cost of gluten-free food. These 2 participants discussed severe financial impacts of their CD prior to diagnosis as a result of stopping working due to their ill health, with 1 male participant explaining:

Fourteen years undiagnosed coeliac, 2 to 3 years towards recovery, [um] that’s seventeen years […] run on impact on pension […] impact on family, on the children, on their education and so on. [Participant 13]

### Development of candidate items

**Figure 1:**
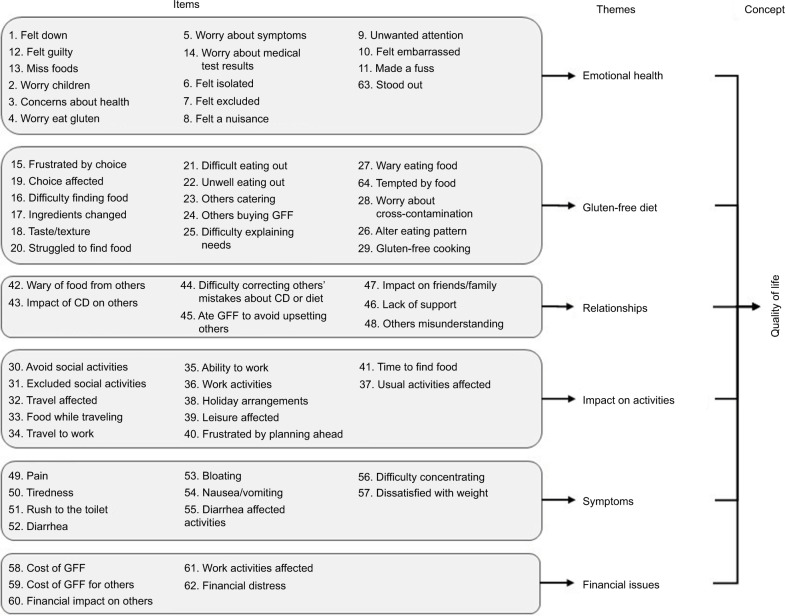
Conceptual framework of quality of life and associated themes and candidate items for CD. **Abbreviations:** CD, coeliac disease; GFF, gluten-free food.

The 6 themes identified from the qualitative analysis constitute the conceptual framework. Sixty-four candidate items (9 for symptoms, 15 for emotional health, 16 for gluten-free diet, 7 for relationships, 12 for impact on activities, and 5 for financial issues) were developed to form the first draft of the Coeliac Disease Assessment Questionnaire (CDAQ). Items were developed to reflect the subthemes of each of the 6 main themes. Generally, more than 1 candidate item was developed for each subtheme, to allow multiple items representing similar issues to be pretested in the next phase of the development of the measure. Figure 1 shows the item content for each candidate item developed within every theme. One aspect that was identified as important in the qualitative interviews as being impacted by CD was holidays. This was a challenging item to include in a PROM, as holidays do not occur on a regular basis and the time frames for PROMs tend to be relatively short (ie, 4 weeks). This issue was addressed as part of a “travel” item, which could refer to holiday travel but also daily travel to work. Some participants also expressed a concern that their children or grandchildren may develop CD. This was not suitable for inclusion as some people do not have children or grandchildren. Therefore, the item was worded as concerns about family members.

Additionally, the response scale was selected, ie, a 5-point frequency scale ranging from “never” to “always”. This is a widely used response set in PROMs. The recall period was set to 4 weeks to capture the full impact of living with CD. The qualitative interviews highlighted that CD can fluctuate according to periods of gluten consumption or social activities and hence a 4-week time period over which respondents can average their responses was thought to provide a good estimate of these fluctuations.

## Discussion

This study investigated how adults with CD perceive the impact of their condition on HRQoL in view to develop candidate items for a new PROM. To comply with FDA guidance,^17^ a new PROM needs to be developed on the basis of qualitative interviews. This step ensures the content validity of the new measure. Hence this study has been conducted to comply with this requirement for the development of a new valid PROM. The interviews with 23 participants revealed 6 main themes that were impacted including symptoms, gluten-free diet, emotional health, impact on activities, relationships, and finances. The symptoms are at their most severe prior to commencing the gluten-free diet, but they can continue following treatment, in particular when there is very high gluten sensitivity, problems with adherence to the diet, or cross-contamination, and therefore, remain an important aspect of QoL in this population. Not all existing PROMs include a dimension on symptoms, eg, CD-QoL,^15^ but this study has highlighted that it is an important part of QoL in CD.

Despite the gluten-free diet being known to improve QoL in CD, as measured by the EuroQol 5 dimension (EQ-5D) questionnaire, a generic measure of HRQoL,^26^ it is also known that the gluten-free diet is burdensome,^1^ restrictive, and challenging to adhere to.^2^,^3^ This study highlights further the impact that the gluten-free diet has on participants and the challenges that people with CD face to adhere to the diet. A Swedish qualitative study on the lived experience of CD similarly found that CD and the gluten-free diet could lead to people feeling “controlled by food” with impacts on work, shopping, travel, and meals at home and away from home.^7^ QoL is worse, and the likelihood of depression or anxiety is higher with increased perceived difficulty of the diet,^12^ hence it is important that PROMs specifically developed for people with CD include items on the impact of the gluten-free diet.

Additionally, the participants described an impact on their emotional health including worries about family, feeling embarrassed by the condition, and feeling isolated. A review recently concluded that CD has a considerable psychological impact, and the gluten-free diet may be 1 of the contributing factors to this impact.^27^ Similarly, impact on activities has been described previously, with travel and dining out affected^28^ as well as impacts on relationships with people with CD reporting unwanted visibility, neglect, and being forgotten.^7^ A negative impact of CD on family life has also been described.^28^

While there is some evidence for the majority of the themes identified in this study, less is known about the financial impact of CD and following a gluten-free diet. It is known that the diet is more expensive than consuming a gluten-containing diet, and the availability of gluten-free food can be limited;^3^,^29^–^31^ less is known about the impact that this higher cost has on people with CD. This may be of particular relevance for people on lower incomes, and if adherence to the diet is hampered by the high costs of the food, this may have a considerable impact on QoL.

The 64 items developed constitute the first (long) draft of the CDAQ. The items reflect all the issues of importance to people with CD. In the next phase of the work, these items were pretested and refined to lead to a shorter draft version of the CDAQ. This phase involved feedback from people with CD and clinicians (including gastroenterologists, dietitians, and Coeliac UK). Subsequently, the draft CDAQ was administered in a survey to produce the final version with its subscales. The data collection and analyses have been completed on these phases and are currently being written up for publication.

Some limitations of the study need to be acknowledged. Despite efforts, low number of ethnic minorities was recruited. The validity of the items for ethnic minorities can be tested in further studies. Second, the participants were recruited through Coeliac UK, a patient charity, which may raise concerns that the sample is biased. There is no evidence that Coeliac UK members are different to people with CD recruited through coeliac clinics or that their experience of QoL issues in CD is different from those who are not a member of Coeliac UK. Usually in England, coeliac clinics are aimed at people seeking a diagnosis or newly diagnosed and, as such, would not have constituted a broad enough sample. However, as Coeliac UK members are likely to be engaged and informed people with CD, they may be in a position to provide richer data on issues of importance in CD and that would constitute an advantage for a qualitative study seeking richness of data. Third, the majority of the participants (15/23) were aged 65 years or older. The most common age of diagnosis is in the fourth to sixth decades,^32^ and the prevalence is higher in older people;^25^ therefore, it was not unexpected that a larger number of participants were older. However, for the later interviews, younger people were specifically recruited to ensure that there were no differences in issues of importance to younger people with CD. The recruitment of younger people did not have an impact on data saturation, which was achieved by participant 8.

Current PROMs are limited as they do not include all the dimensions of QoL of relevance to people with CD (eg, they either only focus on symptoms or have not included symptoms) or they have not been developed according to best standards for PROM development such as FDA guidelines.^17^ Although multiple instruments can be used in conjunction, this can place undue burden on participants. The CDAQ, once fully developed, will have the advantage of being a single instrument able to assess a wide spectrum of issues that are important to people with CD.

## Figures and Tables

**Table 1:** Topics discussed in the semistructured interviews

Tell me about when you first suspected that something was wrong?
Can you tell me about the process that you went through to get your diagnosis?
How does coeliac disease impact on your life?
How have your friends and family reacted to your diagnosis?
How, if at all, has coeliac disease impacted on your emotional health or well-being?
What are your experiences in needing to follow a gluten-free diet?
How would you describe living with coeliac disease to somebody who has just been diagnosed?

**Table 2:** Demographic and disease characteristics of interview participants

ID	Age	Gender	Marital status	Ethnic origin	Occupational status	Duration since diagnosis (years)
1	82	M	Married	White British	Retired	40
2	90	M	Married	White Irish	Retired	35
3	87	M	Married	White British	Retired	2
4	71	F	Married	White British	Part-time work	7
5	77	M	Married	White British	Retired	9
6	75	F	Married	White British	Retired	10
7	66	M	Married	White British	Part-time work	15
8	59	F	Widowed	White Irish	Unemployed	11
9	77	F	Widowed	White British	Retired	35
10	76	F	Divorced	White British	Retired (volunteer)	18
11	89	M	Widowed	White British	Retired	6
12	77	F	Divorced	White British	Retired	8
13	70	M	Married	White British	Retired	13
14	46	F	Married	White British	Full-time work	2
15	65	F	Married	White British	Part-time work	30
16	66	M	Married	White British	Part-time work	5
17	69	F	Married	White British	Retired	10
18	70	F	Married	White British	Retired (volunteer)	7
19	37	F	Married	White British	Full-time work	37
20	38	F	Married	White British	Part-time work	6
21	29	F	Married	White British	Full-time work	1
22	48	F	Married	Asian/British Indian	Part-time work	6
23	32	F	Single	White Irish	Full-time work	<1

**Abbreviations:** CD, coeliac disease; F, female; M, male.

**Table 3:** Qualitative interview themes and subthemes

Symptoms
Concentration
Energy
Gastrointestinal
Pain
Weight
Gluten-free diet
Acceptability of gluten-free food
Cross-contamination
Eating outside of the home
Food choice
Food shopping
Risk
Emotional health
Concerns and worries
Feelings
Isolation and exclusion
Unwanted visibility
Impact on activities
Avoiding social activities
Holidays
Other social activities
Planning ahead
Time
Traveling
Work
Relationships
Lack of understanding
Support
Trust
Financial issues
Cost of gluten-free food

**Table 4:** Data saturation table

Code	Interview (participant ID)																							Number of participants discussing theme
	1	2	3	4	5	6	7	8	9	10	11	12	13	14	15	16	17	18	19	20	21	22	23	
Symptoms																								
Gastrointestinal	**X**	X			X		X		X	X	X	X	X	X	X	X	X	X	X			X	X	17
Weight	**X**	X	X				X		X	X		X	X	X	X			X	X			X		13
Energy				X			**X**			X		X	X	X	X		X							8
Pain					**X**		X		X	X	X		X										X	7
Other symptoms	**X**	X	X	X	X		X			X	X		X	X	X		X		X			X		14

Gluten-free diet																								
Acceptability of gluten-free food	**X**	X	X	X	X			X			X	X	X	X	X	X	X	X	X			X		16
Cross-contamination	**X**		X	X				X	X	X	X	X		X	X	X	X	X	X	X	X	X	X	18
Risk		**X**	X		X		X		X		X				X				X			X		9
Dietary adherence	**X**	X	X	X	X		X	X	X	X	X	X	X	X	X	X	X	X	X			X	X	20
Eating outside of the home	**X**	X	X	X	X	X	X	X	X	X	X	X	X	X	X	X	X	X	X	X	X	X	X	23
Food choice	**X**	X	X	X	X	X	X	X	X	X	X	X		X	X	X	X		X	X		X	X	20
Food shopping	**X**	X	X	X	X	X	X	X	X	X	X	X		X	X	X		X	X	X	X	X	X	21

Emotional health																								
Feelings	**X**	X	X	X	X	X	X	X	X	X	X	X	X	X	X	X	X	X	X	X	X	X	X	23
Concerns and worries	**X**	X	X	X	X		X	X	X	X	X	X	X	X	X	X	X	X	X	X	X	X	X	22
Isolation and exclusion			**X**					X		X			X	X	X							X		7
Unwanted visibility	**X**			X	X		X	X	X	X	X	X		X				X	X	X	X			14

Impact on activities																								
Holidays	**X**	X	X	X	X	X		X	X	X	X	X	X	X	X	X	X	X	X	X	X	X	X	22
Traveling				**X**	X				X		X	X	X	X	X				X	X		X		11
Work	**X**	X		X	X		X	X	X				X	X	X		X	X	X	X	X	X	X	17
Planning ahead			**X**	X	X			X	X		X	X		X	X		X	X	X	X	X	X		15
Other social activities	**X**	X			X					X		X		X	X		X	X	X	X	X			12
Avoiding social activities				**X**	X		X		X			X		X	X			X	X			X		10
Time							**X**			X			X					X	X			X		6

Relationships																								
Lack of understanding from others					**X**	X		X	X	X			X	X	X		X	X	X	X	X	X		14
Support	**X**	X	X	X	X			X	X	X	X	X	X	X	X	X	X	X	X	X	X	X	X	21
Trust	**X**		X		X		X	X		X		X	X		X	X		X	X	X	X			14

Finances																								
Impact on finances	**X**	X	X	X	X		X	X	X			X	X	X	X		X		X	X		X	X	15

**Notes:** X indicates that the participant spoke about the subtheme. **X** indicates the first time the issue was raised.
